# 3D Imaging of Indentation Damage in Bone

**DOI:** 10.3390/ma11122533

**Published:** 2018-12-13

**Authors:** Tristan Lowe, Egemen Avcu, Etienne Bousser, William Sellers, Philip J. Withers

**Affiliations:** 1 Henry Moseley X-ray Imaging Facility, Henry Royce Institute, School of Materials, The University of Manchester, Manchester M13 9PL, UK; egemen.avcu@manchester.ac.uk (E.A.); etienne.bousser@manchester.ac.uk or etienne.bousser@polymtl.ca (E.B.); p.j.withers@manchester.ac.uk (P.J.W.); 2 Ford Otosan Ihsaniye Automotive Vocational School, Machine and Metal Technologies, Kocaeli University, 41680 Kocaeli, Turkey; 3 Engineering Physics Department, Polytechnique Montréal, Montreal H3T1J4, QC, Canada; 4 School of Earth and Environmental Sciences, The University of Manchester, Manchester M13 9PL, UK; William.Sellers@manchester.ac.uk

**Keywords:** aging, in situ, crack initiation and propagation, damage modes, osteoporosis, osteogenesis imperfecta, porosity, bone matrix quality

## Abstract

Bone is a complex material comprising high stiffness, but brittle, crystalline bio-apatite combined with compliant, but tough, collagen fibres. It can accommodate significant deformation, and the bone microstructure inhibits crack propagation such that micro-cracks can be quickly repaired. Catastrophic failure (bone fracture) is a major cause of morbidity, particularly in aging populations, either through a succession of small fractures or because a traumatic event is sufficiently large to overcome the individual crack blunting/shielding mechanisms. Indentation methods provide a convenient way of characterising the mechanical properties of bone. It is important to be able to visualise the interactions between the bone microstructure and the damage events in three dimensions (3D) to better understand the nature of the damage processes that occur in bone and the relevance of indentation tests in evaluating bone resilience and strength. For the first time, time-lapse laboratory X-ray computed tomography (CT) has been used to establish a time-evolving picture of bone deformation/plasticity and cracking. The sites of both crack initiation and termination as well as the interconnectivity of cracks and pores have been visualised and identified in 2D and 3D.

## 1. Introduction

Bone is one of the major innovations of vertebrates, allowing a rigid endoskeleton that can both grow to a genetically controlled shape and size and also remodel dynamically in response to load. Structurally it is a composite material consisting of high stiffness, but brittle, crystalline bio-apatite combined with compliant, but tough, collagen fibres [1]. Physiologically, it accommodates growth, remodelling, repair, metabolism and sensation. The material components are arranged in a highly organised fashion with repeated and nested functional units. The structure of bone has evolved to provide the necessary rigid framework for effective locomotion as well as the protection of vital organs [2]. Typical limb bones have an overall shape reflecting the joints and muscle attachment points with an internal structure consisting of spongy trabecular bone at the epiphyses and metaphysis and forming a rigid, hollow tube of cortical bone in the shaft. Within the cortical bone lies an organised microstructure of laminated bone forming multi-layer, multi-scale tubes which allow the physiological functions of bone to occur alongside its biomechanical role. However, this microstructure varies between individual bones, evolves with age, and differs between different vertebrate species [3]. Bone is highly mass optimised, with peak strains due to normal activity that are approximately half the yield strain, providing a safety factor of about two according to strain gauge studies [4]. Unfortunately, bone fractures in humans are all too common, particularly in aging populations where falls are more frequent and bone quality is lower. Conditions that reduce bone loading, such as bed rest, spaceflight, and a reduction in physical activity associated with growing old, lead to a weakening of the bone due to remodelling that results in an increased risk of traumatic injury [5].

Indentation techniques have become some of the most widely used methods to characterise the fracture behaviour of bone tissue over the last decade [6,7,8]. These techniques allow for the determination of mechanical properties such as hardness and elastic modulus via the acquired load-displacement curves [9]. Since indentation testing can be applied at different length scales to evaluate the mechanical resistance of bone to plastic deformation [8], it has a great potential for investigating and assessing bone quality, plasticity and fracture properties [10,11]. Bone fails due to cracks that propagate through the crystalline matrix. However, the microstructure of bone is such that crack propagation is often stopped at an early stage, and these micro-cracks can be quickly repaired by the body’s internal repair processes [12]. It is only in situations where cracks can propagate through most of the shaft, either because of a succession of small injuries without sufficient time for repair (so-called fatigue fractures [13]), or because the traumatic event is sufficiently large to overcome the individual crack blunting mechanisms, that these cracks lead to catastrophic fracture [14,15]. Thus, a detailed understanding of crack propagation within bone is essential to better understand the process of bone fracture, and the risk factors associated with developmental processes, disease and activity. As such this area of biology has been intensely studied over the last few decades. The advent of high-resolution CT has allowed cracks and microcracks to be accurately visualised and quantified (e.g., [16,17]), however previous work in this area has concentrated on visualising cracks after they have formed. There has been very limited work on time-lapse imaging of loaded bone [18] to measure and track crack growth directly. As a result, considerably more work is merited, particularly with regard to indentation methods that could potentially allow in vivo assessment of bone quality. Dynamic visualisation of a crack whilst it is forming requires both high resolution to allow the crack tip to be adequately imaged, and also fast acquisition rates to allow multiple scans to be taken over many loading increments, whilst minimising time-dependent creep. It also requires specialised loading equipment to apply the load or strain without unduly obstructing the X-rays within the scanner. On the one hand, recent advances in laboratory-based micro CT imaging mean that, from a spatial resolution viewpoint, a synchrotron is no longer necessary for much of the imaging. On the other hand, for time-lapse experiments, synchrotrons significantly outperform laboratory-based systems in terms of acquisition rate and signal to noise [19,20,21]. Nevertheless, limited access to synchrotron beamtime means that if X-ray imaging is to become a useful research tool in the study of bone mechanics as a function of aging, diet, osteoporosis or repair, then it is important to consider laboratory-based X-ray CT methods.

The purpose of this study is to examine what can be achieved using state-of-the-art X-ray CT systems and customised indentation rigs in terms of visualising and tracking bone plastic deformation and crack propagation under indentation loading. This study questions whether laboratory-based systems are adequate for applications such as non-linear bone fracture modelling, bone property measurement, and the experimental identification of bone plasticity and toughening mechanisms. 

## 2. Materials and Methods 

**Figure 1:**
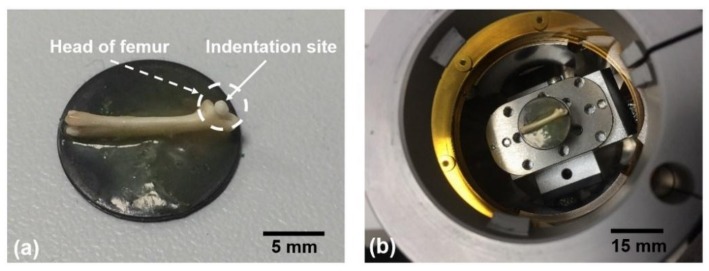
(**a**) Bone mounted on a steel plate with the femoral head pointing vertically for indentation, (**b**) Specimen mounted within the Hysitron in-situ nanomechanical testing rig.

A dried, de-fleshed mouse femur was chosen for this study (supplementary document S1): it is a suitable size to fit within the available chambers; the current rig is not humidity controlled so a wet specimen would not be suitable, and the mouse is a commonly used species for studying bone physiology and pathology [22]. The specimen was mounted on a magnetic plate using wax to ensure the head of the femur was positioned vertically for indentation of the fine trabecular region (Figure 1).

**Figure 2:**
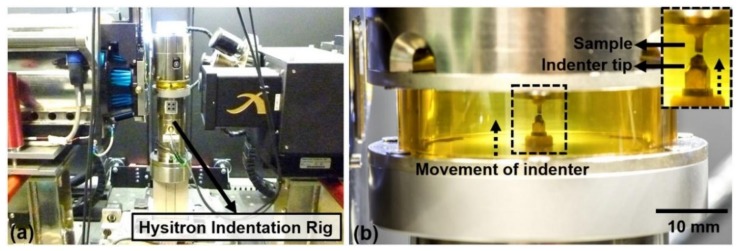
(**a**) The Hysitron indentation rig mounted within the Zeiss-Xradia VersaXRM-520 system during initial specimen alignment, (**b**) Magnified image showing the indentation tip position.

Indentation was performed using a specially designed Hysitron in-situ nanomechanical testing rig (Bruker Hysitron IntraSpect 360 indentation rig) developed in association with the Henry Moseley X-ray Imaging Facility. It was mounted in a Zeiss Xradia 520 Versa X-ray microscope system (Figure 2) to allow imaging while the indentation was taking place. The Hysitron rig uses a piezoelectric load cell design with capacitive depth sensing. It can provide a maximum force of 10 N and maximum displacement of 80 μm and will run in load- or displacement-controlled testing modes using a Performech digital controller. This allows it to capture transient events, such as fracture initiation, whilst using a wide variety of probe materials and geometries. The indentation was performed using a three-sided pyramidal (Berkovich) indenter under load-controlled indentation mode. The Zeiss Xradia 520 Versa was operated at 110 kV and 91 mA with a specimen-to-source distance of 94 mm and a specimen-to-detector distance of 43.4 mm for optimum imaging geometry to optimise the resolution of the images obtained by minimising both pixel size and focal spot blurring [23]. The whole indentation area could be viewed at 9.77× magnification corresponding to a voxel size of 0.95 µm under these settings. Each projection was collected over 120 s to minimise image noise in the projections and maximise spatial resolution, resulting in a 2D spatial resolution in the 2–3 µm range which was validated using a JIMA spatial resolution chart. The specimen was rotated over a 360° rotation range, collecting 1601 projections and giving an overall scan time of 52 h.

After image acquisition, the data sets were uploaded into the Zeiss Xradia XMReconstructor software for reconstruction of the 3D virtual slices using a filtered back projection algorithm. The reconstructed data was then analysed using the Avizo 9.2 (Thermo Fisher Scientific, Waltham, MA, USA) visualisation software to segment and image the virtual slices and 3D volume renderings. After the indentation experiment, the residual imprint location on the femoral head was investigated using an FEI Magellan scanning electron microscope (SEM), and secondary electron (SE) images of the indentation site were collected at 1 kV with a tilt angle of 45°. 

## 3. Results and Discussions

**Figure 3:**
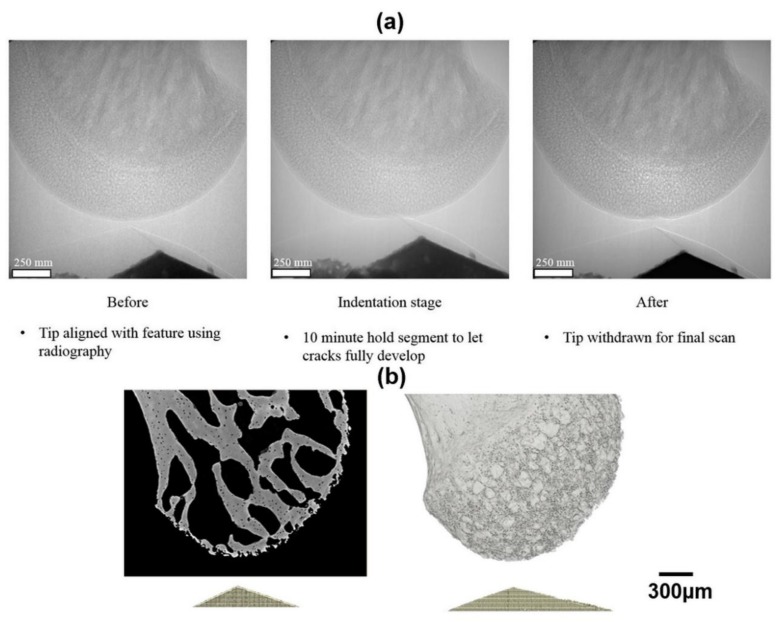
(**a**) Radiographs showing the indentation sequence using a Berkovich indenter geometry, (**b**) Virtual slice and 3D reconstruction of the head of the mouse femur.

**Figure 4:**
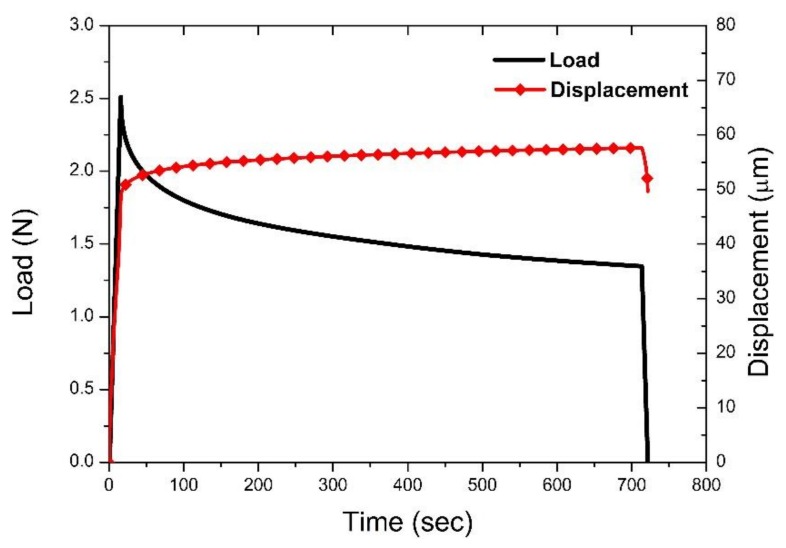
The load and displacement curves recorded as a function of time.

The indentation sequence is illustrated in Figure 3a with the load and displacement curves recorded shown in Figure 4. The load was linearly incremented from 0 to a maximum of 2.5 N, since at this point the displacement sensor limit was reached and the sensor saturated, leading to a significant drop in load and a slight increase in displacement during the hold segment (Figure 4).

**Figure 5:**
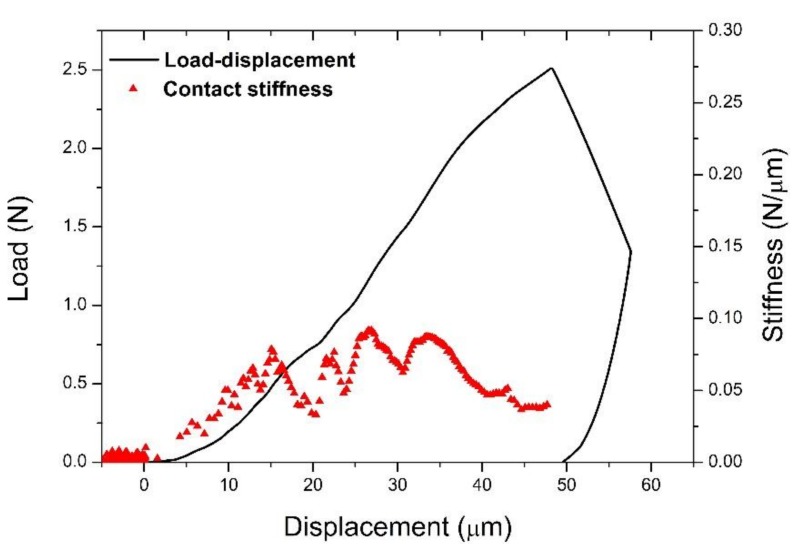
The load-displacement curve of the full indentation and variation of the contact stiffness during the loading segment. The load drop during the hold stage is evident.

**Figure 6:**
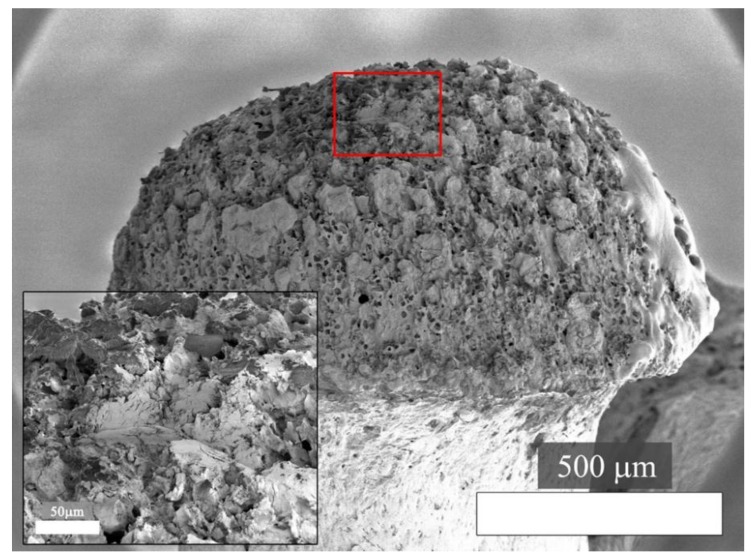
Secondary electron SEM image of the indentation residual imprint location on the femoral head and higher-magnification of the indentation (inset) at 1 kV with a tilt angle of 45°.

The load-displacement curve of the full indentation and the corresponding contact stiffness (dP/dh) during loading are illustrated in Figure 5. The fracture of the bone can be identified in the load-displacement curve through pop-in events in the loading segment, which translate to significant drops in contact stiffness accompanying local fractures (Figure 5). The residual imprint does not have the usual shape for a Berkovich indentation and seems to be slightly elongated (Figure 6). This could indicate the movement of the tip during the indentation. In addition to the sensor saturation effects, this could be one of the reasons for the drop in load and displacement drift during the test holding segment (Figure 4).

Due to the heterogeneous microstructure of bone [24], as illustrated in Figure 3b, the selection of the indentation site is essential [25] in determining the mechanical response and the inferred properties such as hardness and elastic modulus [26,27]. One other factor that needs to be considered in terms of indentation testing is the size of the indenter [28]. The inferred mechanical properties of the bone are likely to depend on the indent size due to the scale of the microstructural features such as pores and cracks within the bone structure sampled during the indentation process [29]. In the present study, the indent size is not a critical concern since the main focus of the study is to generate and visualise sub-surface crack networks to understand the crack propagation and fracturing behaviour of bone rather than to examine the mechanical properties (e.g., hardness and modulus) by implementing indentation under X-ray CT. 

**Figure 7:**
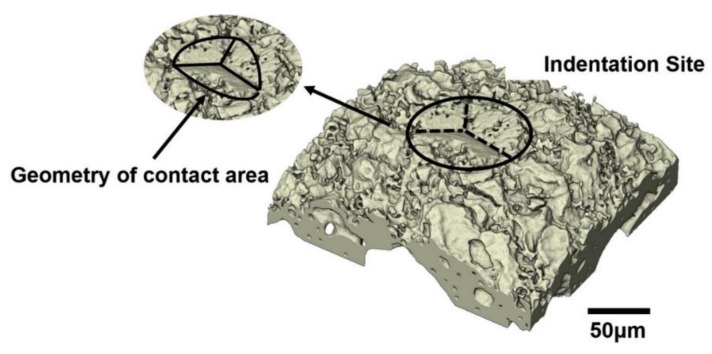
Volumetric rendering of the indentation site and geometry of the contact area after removal of the indenter.

**Figure 8:**
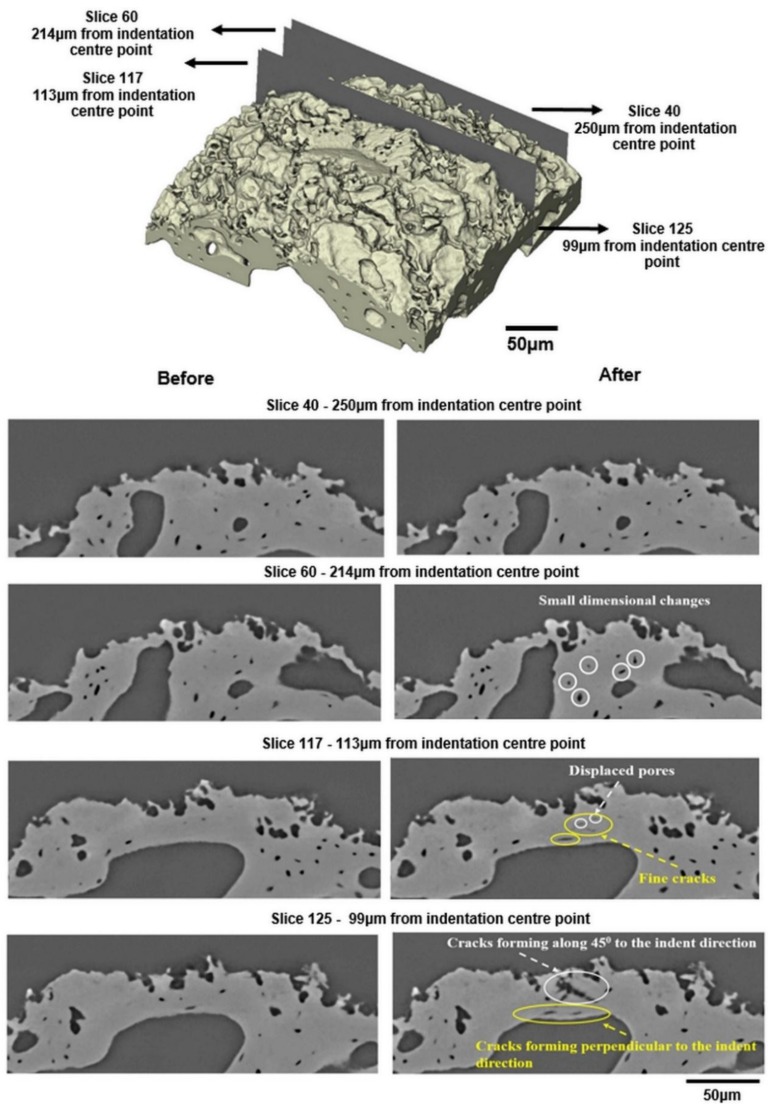
Individual virtual slices before (**left**) and after (**right**) indentation at distances of 250 µm, 214 µm, 113 µm and 99 µm from the indenter tip (see also animated sequences in supplementary video online).

Figure 7 shows a 3D image of the indentation site and also the geometry of the contact area formed during the indentation. This indicates a material pile-up behaviour [30]. It has been reported that pile-up of material on the indentation site may occur when using a sharp Berkovich indenter [9,31,32]. However, the amount of pile-up is limited, as seen in Figure 7, since bone has a relatively low effective modulus-to-yield stress ratio in compression [32]. Although the plastically deformed indentation site and limited pile-up can be seen through the volumetric rendering of the indentation site given in Figure 7, it is not possible to identify the sub-surface damage mechanisms such as splitting, cracking and plastic deformations induced by the indentation. Therefore, individual virtual cross-sectional slices (orthoslices) at different distances have been used to identify these damage modes and to understand the fracture mechanics of bone. This is challenging due to the complex and hierarchical structure of bone [33]. Figure 8 shows the individually rendered cross sections at large distances away from the indentation site and near the indentation site. Small dimensional changes through the movement of small pores in the bone microstructure are visible at a distance of 214 µm from the indentation site (slice 60) while no visible changes can be observed at a distance of 250 µm (slice 40). 

Figure 8 shows various damage mechanisms including visible plastic strain and crack initiation and propagation through the virtual cross-sectional slices near the indentation site (slices 117 and 125). Fine cracks and the displacement of pores can be identified at a distance of 113 µm (slice 117) while crack formation and growth at an angle of approximately 45° to the indentation direction can be observed nearer to the indentation site (slice 125). Crack bridging is one of the major mechanisms that inhibits crack extension in bone tissue [34] and can occur when a crack grows from the indent [27]. Here the cracks along the direction 45° to the indentation direction show some slight evidence of crack bridging (Figure 8 slice 125) which may lead to a decrease in the driving force at the crack tip through the interaction between the cracks and the microstructural features [35]. Some interactions between the propagating cracks and the pre-existing pores and micro-cracks are also visible in slices 117 and 125. Although it is clear that the pore networks in the bone structure affect the mechanical behaviour of bone [36], it is currently unknown how important these pores are in preventing crack propagation and increasing the fracture resistance by contributing to toughening mechanisms such as microcracking, crack bridging, and crack deflection [10,15,16,35]. Haversian canals have an important role in the crack propagation behaviour of human cortical bone (specifically crack deflection) [15], however, they have been reported as ‘not found’ in mouse bone, and in any case not in trabecular bone [7]. Thus, the interconnectivity of pores and cracks may have a relatively strong effect on the fracturing of mouse bone compared to human bone. In order to better understand the effects of the pre-existing pore networks on the crack propagation, the interaction of cracks with pores and fine trabecular structure is visualised and discussed in detail through 2D and 3D visualisations in the following sections.

**Figure 9:**
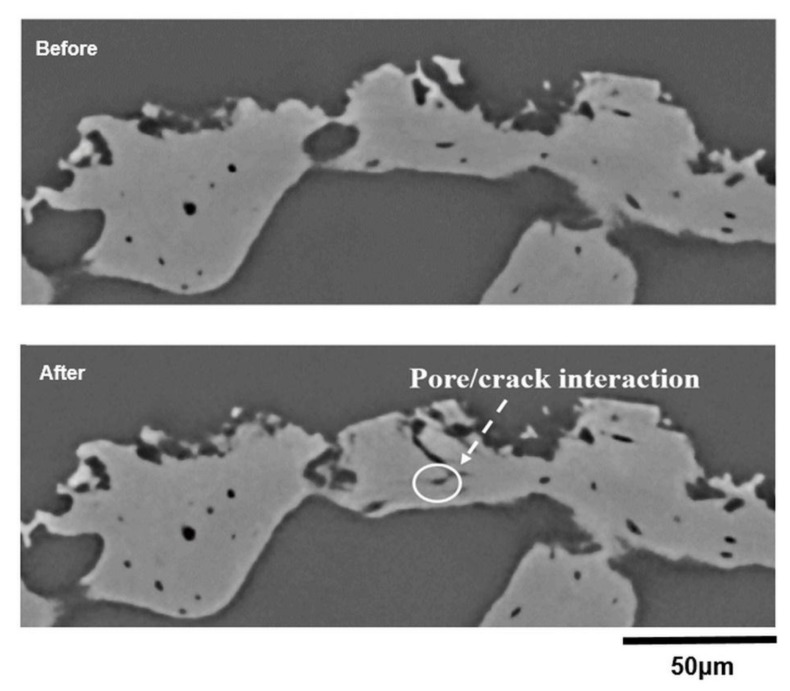
Individual slice images before and after indentation showing plastic deformation, collapse and cracking as a result of the indenter at a distance of 66 µm from the indenter tip (see also animated sequences in supplementary video online).

The propagation of micro-cracks local to the indentation site and the interaction between the growing cracks and the pores found in the trabecular bone can be seen in Figure 8 and Figure 9. It is evident that there is no significant orientation of the pore structure, and the sizes of pores range between 5 and 30 µm. The micro-crack formation might be dependent on these pre-existing pore networks illustrated in both Figure 8 and Figure 9. On the one hand, pores may detrimentally affect the mechanical properties of bone due to the decrease in the total load bearing area [37] as well as acting as stress concentration sites. On the other hand, the internal pore structure may have a positive effect on suppressing crack propagation during indentation. Thus, it is important to understand the effects of crack-pore interactions on the toughening mechanisms of bone structure. Unfortunately, the existing literature is limited regarding the analysis of the interaction of cracks with the existing pore networks in the bone microstructure, and no work has been conducted on how cracks develop and interact with the microstructural features of bone over time during indentation loading.

**Figure 10:**
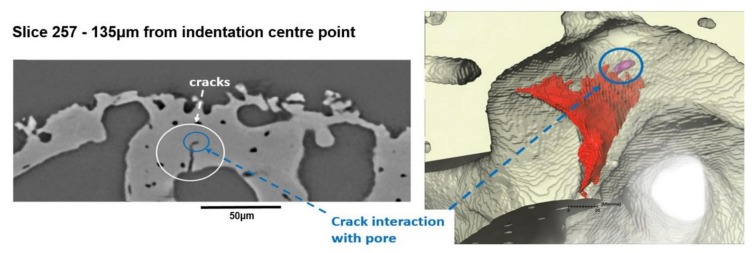
2D and 3D visualisations of a larger flexural crack from the opposite side of the indenter tip showing how the 2D image can be misleading in terms of crack complexity and propagation.

In Figure 9, we can see how a crack interacts with an existing pore within the bone structure, and the growth of the crack appears to be terminated at this point. However, the 2D view of cracks and their interactions with the pores illustrated in these figures can be highly misleading. In fact, the cracks themselves have complex 3D shapes that propagate through the bone, and the level of detail is therefore clearly necessary if this approach is to be used to validate non-linear crack growth models and their complex interactions with the pore networks in the bone structure. Figure 10 illustrates the individually rendered slice at a distance of 135 µm from the opposite side of the indenter tip and the 3D rendering of the crack structure illustrating the interaction between the growing crack and the pores found within the trabecular bone. The 2D visualisation suggests that the pore structure has completely stopped the crack from propagating, but the 3D view showing a significant flexural crack and its interaction with an existing pore implies that the real story is much more complex (Figure 10). To the best of our knowledge, this is the first time that indentation of bone has been visualised in high resolution to analyse the initiation and propagation of cracks and their interactions with the microstructural features of bone, specifically with pre-existing pore networks.

**Figure 11:**
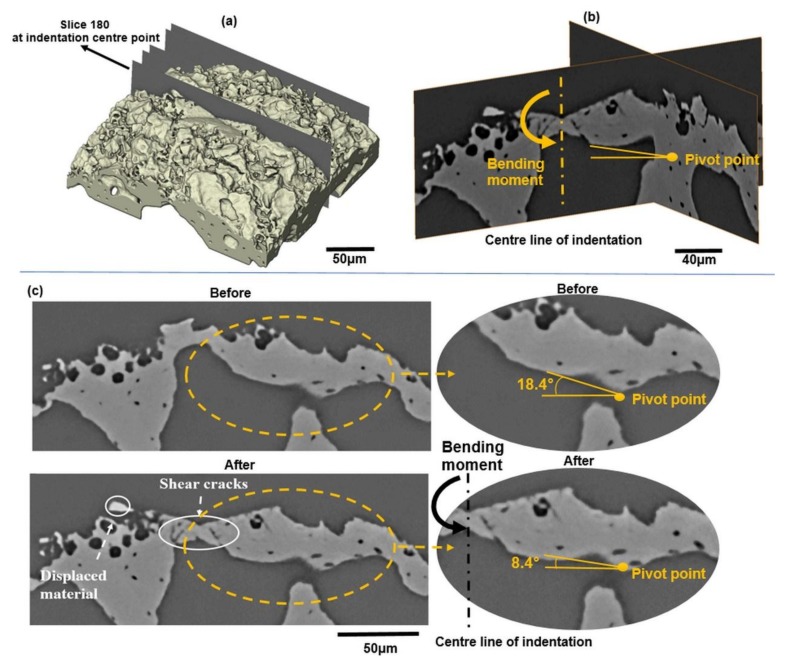
(**a**) Volumetric rendering of the indentation site, (**b**) Orthoslice from the other orientation showing the pivot point, (**c**) Individual slice images showing the damage immediately below the indentation tip (see also animated sequences in supplementary video online).

Figure 11 shows the damage at the centre of the indentation site. Some evidence of plastic deformation beneath the surface is visible which could provide resistance to crack propagation by blunting the crack tip through the formation of plastically deformed zones [7]. However, this intrinsic toughening mechanism seems to be very limited, preventing the formation of large shear cracks just beneath the vicinity of the indent corner (Figure 11c). The rotation of the indentation site around the pivot point (rotation point) is identified through the angular movement of the indented microstructure as highlighted in Figure 11b,c. It can be inferred that the bending moment induced by this identified rotation may be the underlying reason for the severe plastic deformation and shear crack propagation at the centre of the indentation site. A limited pile-up of material can be seen at the right edges of the impression, proving that plastic deformation occurred during indentation while displacement of materials is visible at the left edge due to the generated high strain at the surface of the indentation zone (Figure 11). 

Previously indentation studies have yielded only information on the surface crack geometries, while sub-surface crack behaviour has only been predicted, or investigated through cross-sectional scanning electron microscope (SEM) images [27]. Here we have successfully visualised and linked different surface and sub-surface damage mechanisms in the bone structure, such as localized plastic deformation, pile-up of materials and propagation of different crack types by fracturing the head of a mouse femur using load-controlled indentation testing within the X-ray CT scanner. Since mouse bone has been widely used as a model for human bone diseases [7,24] this approach provides a novel way of exploring the bone quality and the fracture mechanics of bone as a function of aging and/or bone disease. While we are currently limited to using dried bone, future work will include the incorporation of a humidity controlled chamber so that freshly prepared bone can be used, allowing its well known differences in material properties to be assessed [38]. 

## 4. Conclusions

The present paper demonstrates the use of an indentation rig within an X-ray CT scanner to visualise and characterise the cracking of bone material at an unprecedented level of detail for the first time. This has revealed a range of surface and sub-surface damage mechanisms in the bone structure, such as localised plastic deformation and the propagation of different types of cracking. The results show that indentation coupled with X-ray CT has the potential to quantify the bone fracture mechanics associated with aging and disease process, such as osteoporosis and osteogenesis imperfecta. 

The interactions of cracks with the existing pore networks and micro-cracks have been visualised in high resolution in 2D and 3D to understand the fracturing process of the bone structure. Further research is recommended on the 3D visualisation of these interactions to understand the fracture mechanics of bone. Time-lapse 3D imaging is a promising tool to better understand in-vivo indentation of bone to evaluate bone quality, the effects of normal development, and the impacts of disease processes and potential treatments.

The results illustrate that the 3D propagation of cracks and other deformation effects within the bone under the indentation load can be visualised with a resolution of ~2 µm by laboratory-based X-ray CT. This is sufficient to observe early crack formation while enabling us to see the microstructural features that might help prevent crack propagation. However, the current acquisition rate means that each scan took 52 h to acquire. This limited the number of frames that could be acquired in a time-lapse sequence. Furthermore, it introduces the risk of creep and relaxation affecting the interpretation of the results. Therefore, in-situ indentation tests under synchrotron X-ray CT imaging would be desirable to observe in-situ crack development as the indenter is progressively loaded. This would also allow the use of CT data to better understand the strain-strain features that occur during indentation in terms of the plastic hinging and crack propagation sub-surface. Such an approach will enable better interpretation and of indentation curves in terms of the effects of aging or disease. Nevertheless lab. X-ray CT is demonstrated to be a useful tool for characterising and quantifying sub-surface indentation damage for bone indentation testing.

## Supplementary Materials

The following are available online at http://www.mdpi.com/1996-1944/11/12/2533/s1, Video S1: Animated sequences showing indentation damage in bone, Document S1: Materials and methods.
