# Supplementary material for: 3D Imaging of Indentation Damage in Bone
Source: Materials (Basel). 2018 Dec 13;11(12):2533. doi: 10.3390/ma11122533 (PMC6316674; doi:10.3390/ma11122533)
Supplement: Supplementary file 1 [file materials-11-02533-s001.zip › Document S1 (materials and methods).pdf]

materials

Supplementary Materials (Document S1)

3D Imaging of Indentation Damage in Bone

Tristan Lowe[1,¶,*], Egemen Avcu[1,¶,2], Etienne Bousser[1,3], William Sellers[4] and Philip J. Withers[1]

[1] Henry Moseley X-ray Imaging Facility, Henry Royce Institute, School of Materials, The University of Manchester, Manchester M13 9PL, UK; egemen.avcu@manchester.ac.uk (E.A.); etienne.bousser@manchester.ac.uk (E.B.); p.j.withers@manchester.ac.uk (P.J.W.)

[2] Ford Otosan Ihsaniye Automotive Vocational School, Machine and Metal Technologies, Kocaeli University, 41680 Kocaeli, Turkey

[3] Engineering Physics Department, Polytechnique Montréal, Montreal H3T 1J4, QC, Canada; etienne.bousser@polymtl.ca

[4] School of Earth and Environmental Sciences, The University of Manchester, Manchester M13 9PL, UK; William.Sellers@manchester.ac.uk

¶ These authors contributed equally to this work.

* Correspondence: Tristan.Lowe@manchester.ac.uk (T.L.); Tel.: +44-161-306-2250

Materials and Methods

The mouse femur was sourced from the University of Manchester osteological reference collection. These are skeletons from cadavers collected in the wild from individuals that have died of natural causes and are used for osteological teaching. The individual chosen was recently collected, adult and with no signs of pathology.
